# Proteomics analysis of chronic skin injuries caused by mustard gas

**DOI:** 10.1186/s12920-022-01328-3

**Published:** 2022-08-06

**Authors:** Vahid Jamshidi, B. Fatemeh Nobakht M. Gh, Shahram Parvin, Hasan Bagheri, Mostafa Ghanei, Alireza Shahriary, Seyyed Masoud Davoudi, Masoud Arabfard

**Affiliations:** 1 Chemical Injuries Research Center, Systems Biology and Poisonings Institute, Baqiyatallah University of Medical Sciences, Tehran, Iran; 2 Education Office, Pasteur Institute of Iran, Tehran, Iran

**Keywords:** Proteomics analysis, Mustard gas, Chemical injuries, Systems biology, Chemical agents

## Abstract

Sulfur mustard (SM) is an alkylating and forming chemical that was widely used by Iraqi forces during the Iran–Iraq wars. One of the target organs of SM is the skin. Understanding the mechanisms involved in the pathogenesis of SM may help better identify complications and find appropriate treatments. The current study collected ten SM-exposed patients with long-term skin complications and ten healthy individuals. Proteomics experiments were performed using the high-efficiency TMT10X method to evaluate the skin protein profile, and statistical bioinformatics methods were used to identify the differentially expressed proteins. One hundred twenty-nine proteins had different expressions between the two groups. Of these 129 proteins, 94 proteins had increased expression in veterans' skins, while the remaining 35 had decreased expression. The hub genes included RPS15, ACTN1, FLNA, HP, SDHC, and RPL29, and three modules were extracted from the PPI network analysis. Skin SM exposure can lead to oxidative stress, inflammation, apoptosis, and cell proliferation.

**Supplementary Information:**

The online version contains supplementary material available at 10.1186/s12920-022-01328-3.

## Introduction

Sulfur mustard (SM) could be a chemical operations agent that causes blisters on the skin and mucous membranes [1]. Quite 90% of patients exposed to dichloroethyl sulfide showed various skin lesions within the damaged areas. Skin may absorb about 20% of this blistering agent during exposure. About 70% of chemicals can accumulate within the epidermis and the rest within the basement membrane and dermis [2]. SM appears to wreck the skin by disrupting cell proliferation [3]. The Skin symptoms of SM exposure include itching, burning, painless erythema or sunburn, hypopigmentation, hyperpigmentation, and melanoma in exposed and unexposed areas [4, 5]. The mechanism of SM skin symptoms is not fully understood. Acute skin symptoms caused by SM can emerge as itching, erythema, blistering and burning sensation reckoning on the dose and duration of exposure [5, 6]. 2 to 24 h after exposure to SM, only a tiny low number of basal cells show nuclear changes like chromatin loss. Hydropic degeneration of the cytoplasm, and liquefactive necrosis of the epithelium has been reported in acute exposure [7]. The blisters usually appear as small vesicles after about 17 h of exposure to SM, but usually heal within three weeks [8]. In mild cases, the skin lesions could also be limited to erythema that darkens in about 15 days, while the superficial layers of the epidermis become scaly without causing an actual skin defect [9]. Histological studies of skin exposed to SM show vasodilation and neutrophil leakage, indicating the assembly of vasoactive mediators and chemical adsorbents within the damaged areas [10]. Interleukins IL-1α/β, IL-6, IL-8, and TNF-α are released shortly after exposure to SM [11]. The NF-қB pathway and mitogen-activated protein kinases are significantly involved in the the regulation of genes encoding inflammatory cytokines after SM injury [12]. Increased formation of reactive oxygen species and decreased glutathione production occur after exposure to SM [13]. Various metabolites are formed following the reaction of SM with glutathione, which may be detected within the urine [14]. NO signaling pathways also are important in modulating inflammation and necrobiosis in sulfur mustard-exposed skin cells [15]. Although vast amounts of knowledge are obtained in previous research to know the pathophysiology of SM toxicity, many questions remain and more research is needed to answer them [16]. Our main goal during this study was to gauge the expression of different skin proteins in chemical veterans exposed to SM and to search out a possible link between common skin problems including pruritus, eczema, atopic eczema, hyperpigmentation and hypopigmentation and melanoma with changes the expression of skin proteins among these patients.

## Materials and methods

### Statement

The research ethics committee of Baqiyatallah University of Medical Sciences has approved the project (Approval ID: IR.BMSU.REC.1398.114). Informed consent was obtained from all subjects, and all methods were carried out in accordance with the relevant guidelines and regulations of REC. All experiments and relevant protocols were performed in accordance with the relevant guidelines and regulations of Baqiyatallah University of Medical Sciences.

### Patients and controls

After filling in the questionnaire and informed consent, ten patients (SM, moderate to severe) and ten controls who were matched in sex and age were included in the study. Sample inclusion and exclusion criteria are stated in Table 1. The patients had medical records and their mustard injuries were confirmed by expert Dermatologists. Skin biopsy was done the same dermatologist from the inner part of the arm near the axillary fossa. The size of sampling was about 2 * 1 cm^2^ and the depth of it was hypodermic fat. After cleaning of the sample from blood, it was inserted it in normal saline and then prepared for laboratory examination.

**Table1:** Inclusion and exclusion criteria of patients and controls

Inclusion criteria
Adult male
Non-smoker
Age between 45 and 60 years A well-documented exposure with SM
Exclusion criteria
Taken corticosteroids or other specific drugs for 2 weeks
Diabetic, Hypertension, Gout, Asthma and Hyperlipidemia
Surgery in the last 3 months
Seasonal allergies in the last 6 months
Treated with antibiotics for 2 weeks

### Sample preparation

Skin samples were resuspended in 800 μl of 1% sodium deoxycholate in 100 mM NH_4_HCO_3_ was added to the samples and vortexed vigorously; the samples were centrifuged at 5000 RPM. The protein solution was lyophilized and covered with parafilm. According to the manufacturer’s instructions, protein concentrations were determined using the BCA assay (CAT NO:23235 Thermo Scientific™). The disulfide bond of cysteine in the protein was reduced with 10 mM dithiothreitol (DTT) at 37 °C for 1 h, followed by alkylation with 20 mM Ioadoacetamid (IAA) for 45 min in a dark place with room temperature. The remaining IAA in the sample was quenched with 10 mM DTT for 15 min in a dark with room temperature. Proteolysis of samples using Lys-C protease (protein to enzyme ratio 100:1) was performed overnight at 28 °C, followed by trypsin digestion (protein to enzyme ratio 100:1) for 6 h at 37 °C. The pH of the sample was adjusted to approximately 3 using a final concentration of approximately 1% trifluoroacetic acid (TFA) and then desalted using a solid phase extraction disc containing styrene–divinylbenzene, and stepped tips (Empore SDBRPS 47 mm Extraction Disc, SUPLCO). Stage tips were self-packed into pipette tips, peptides were bound to the stage tips, washed with 0.2 percent TFA, and lastly eluted with 80 percent acetonitrile: 5% ammonium hydroxide. The peptides were vacuum centrifuged and then reconstituted in 200 mM HEPES pH 8.8 before being quantified using the Pierce quantitative colorimetric peptide assay (CAT NO:23235 Thermo Scientific™).

### Sample labeling

Peptides were labeled with tandem mass tag (TMT) reagent according to the manufacturer's instructions (CAT NO: 90110 Thermo Scientific™). Briefly, anhydrous acetonitrile was added to each vial labeled with TMT, followed by shaking for 5 min and short centrifugation. Aliquots of individual peptide samples were labeled with one of the individual TMT tags (10 tags total). Labeling was performed at room temperature for 1 h with occasional shaking.

To quench excess TMT label in the samples, 5% hydroxylamine was added to each sample, shaken, and incubated at room temperature for 15 min. A label check experiment was performed by mixing 1.5 μl of each individually labeled vacuum-dried TMT sample to ensure that the same amount of total peptide was pooled in all samples prior to sample pooling. Samples were reconstituted in 2 μN, 0.1 μl water and analyzed by LC connected to a mass spectrometer (QExactive, Thermo Fisher, USA). Normalization factors were obtained from label validation experiments, and samples of TMT-labeled peptides were pooled in a 1:1 ratio across all samples and dried under vacuum. Samples were purified by C18 solid phase extraction desalting (SPE, SepPak, Waters) and centrifuged under a vacuum to dryness. The peptide mixture was fractionated into 96 fractions by high pH reverse phase HPLC (HpH), which were then pooled into 17 fractions before LCMS/MS analysis. HpH HPLC fractions of each TMT kit were reconstituted with sample loading buffer (2% acetonitrile, 97.9% water, 0.1% formic acid) and subjected to LC–MS/MS analysis.

### Preparation of the samples for MS analysis

The HpH HPLC fractions of each TMT set were reconstituted with sample loading buffer (2% acetonitrile, 97.9% water, 0.1% formic acid) and subjected to LC–MS/MS analysis.

### LC–MS/MS analysis

#### 1D data dependent acquisition (DDA) of peptides on QExactive Quadrupole-Orbitrap (QE-classic)

TMT-labeled peptide samples were injected into an in-house packed trap column and desalted with loading buffer. The peptide was eluted from the trap into an in-house packed analytical column with linear gradients of mobile phases A and B (mobile phase B (30%) over 110 min at a flow rate of 300 nL/min across the gradient). The eluent from the trap was separated on the analysis column. The column eluate was fed to the ion source of the mass spectrometer. An electrospray voltage of 2.6 kV was applied through the fluid connection upstream of the column. Peptide precursors from 350 to 1850 m/z were scanned at 70 k resolution using a 1 × 106 target AGC. The 10 strongest ions in the previous survey scan were fragmented by high energy collision dissociation (HCD) using a collision energy of 35 normalized with a separation width of 0.7 m/z. For MS/MS analysis, only precursors with charge levels of + 2 to + 4. In the MS procedure, the minimum signal required for the MS2 trigger was 2.5 × 104, the AGC target value for MS2 was 2 × 105, and the maximum injection time for MS2 was 250 ms. The MS/MS scan resolution was set to 70 k. The dynamic exclusion was set to 90 s.

### Protein identification and quantification

The mass spectrometric data files for each sample set were searched using Proteome Discoverer (version 2.1, Thermo Scientific). Uniprot database (181005_UniPr_HUMAN_Revi + Unrevi.fasta, Ref: http://www.uniprot.org) containing 95,106 human proteins including isoforms and unreviewed (Homo sapiens) was used for searching the data. The quantitative ratios were generated using the quantitative values found in channel 126 (control) as the denominator.

### Data processing

**Fig. 1:**
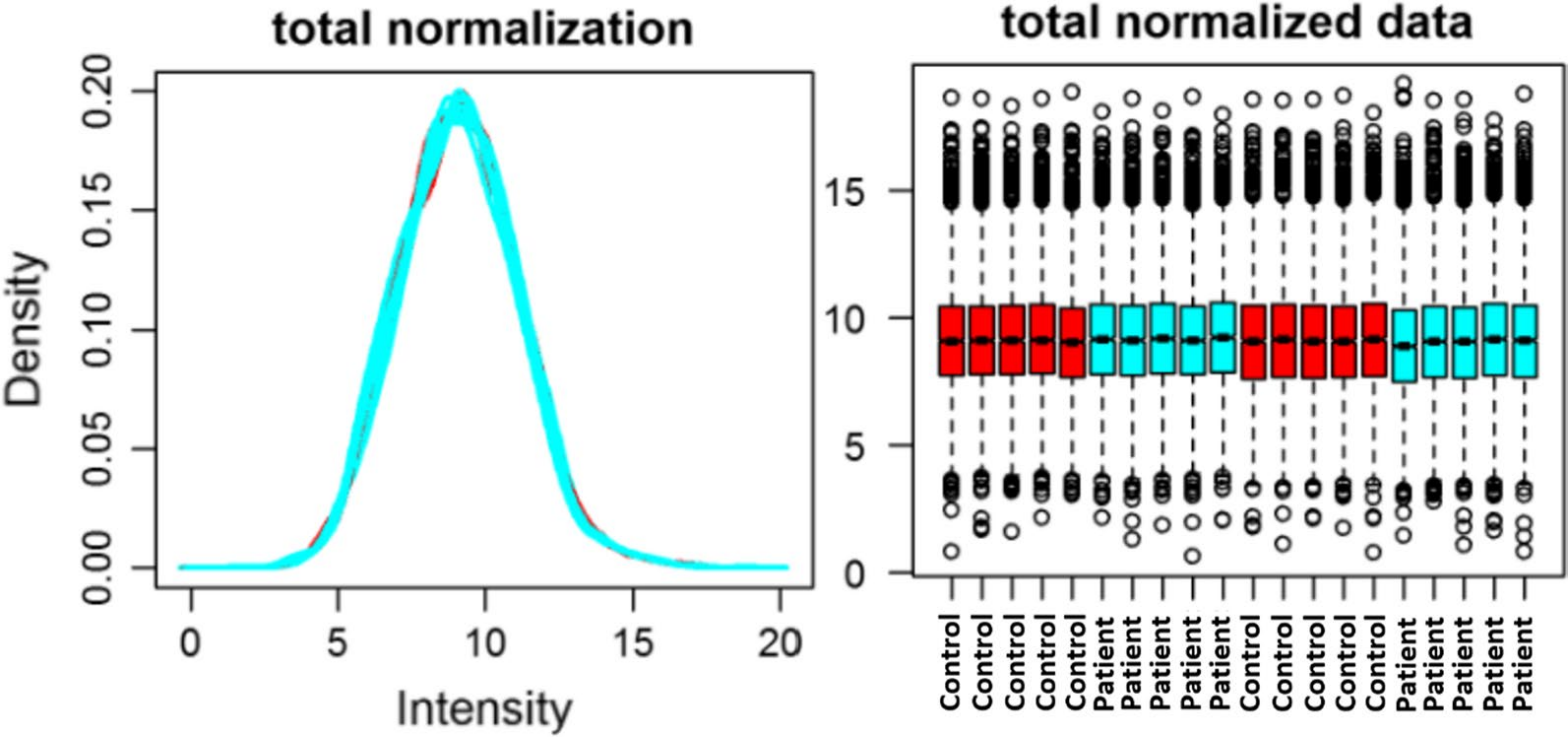
Density plot (left panel) and Box plots (right panel) of post-normalized data showing variability in the TMT-MS data skin

TMT quantitative proteomic analysis of human samples resulted in the identification of 3586 high confident (Protein, Peptide and PSM FDR < 1%) proteins in skin samples. Density plot and Box Plot analysis of normalized data (Fig. 1) show similar protein ratio distribution (ratio with reference to channel 126, a control sample) across all samples within each respective data set, therefore, a relative abundance comparison was performed. Normalization is accomplished by dividing abundance values by the total intensity of the provided array (i.e., the sum of all abundance values).

### Statistical analysis

The differentially abundant proteins were analyzed using the Limma Packages [17], written in R. The Bioconductor software was used to analyze the data, which fits a linear model to each protein and then uses the Bayes technique to the predicted variances to increase power. The results were validated by the ANOVA test separately. Comparison of normalized protein areas between control and patient samples showed a total of 129 differentially abundant proteins (Fold change ≥ 1.2 and Fold change ≤ 0.8, statistically significant differences between patient and control were accepted as *p*-value < 0.05) in skin samples. The reason for choosing this Fold change was due to the chronic nature of the disease. Selecting a higher threshold makes it difficult to find the altered proteins for patients with chronic disease. Moreover, regarding the limited number of samples, the adaptation of Adjusted *p*-value for a low number of samples limits the validation of significant proteins.

### Gene ontology and pathway enrichment

Gene Ontology (GO) was used to define gene functions in biological process (BP), molecular function (MF) and cellular component (CC) aspects. Enrichr is an online enrichment analysis tools that provides a functional annotation set to understand the biological meaning behind a large list of genes or proteins [18]. The functional enrichment analysis of the statistically significant proteins, including GO and KEGG pathway enrichment analysis [19], was conducted using Enrichr.

### PPI network and network analysis

The list of proteins was analyzed using the online website STRING (https://string-db.org/, version 11.5) for protein–protein interaction analysis [20]. Then, the software Cytoscape was used to establish a PPI network. The Network Analyzer in Cytoscape was utilized to calculate node degree [21]. Also, the MCODE plugin was used to perform modular analysis [22], with the parameters set as follows: a Degree Cutoff = 2, Node Score Cutoff = 0.2, K-Core = 2, and Max. Depth = 100.

## Results

### Identification of significant abundance proteins

This study evaluated the protein profiles of 10 healthy individuals and 10 chemical warfare veterans with a history of SM exposure were evaluated. A total of 129 proteins were identified with significant increasing and decreasing regulatory changes, including 35 proteins with down-regulation and 94 proteins with up-regulation (Additional file 1). Table 2 shows the top 10 down regulated skin proteins and Table 3 show the top 10 up-regulated skin proteins.

**Table 2:** Top 10 skin proteins with significant down-regulation in chemical warfare victims

Accession ID	*P*-value	Fold change	Gene name	Protein name	Pathogenesis pathway
P20718	2.5E−03	0.31	GZMH	Granzyme H OS = Homo sapiens OX = 9606 GN = GZMH PE = 1 SV = 1	Due to the fact that granzyme H is primarily expressed in CD3 − CD56 + NK (natural killer) cells of the immune system, decreased expression of this protein is associated with impaired induction of target cell death in pathogen-infected cells
P69891	9.3E−03	0.54	HBG1	Hemoglobin subunit gamma-1 OS = Homo sapiens OX = 9606 GN = HBG1 PE = 1 SV = 2	Dysfunction of blood coagulation, Impairment of cellular oxidant detoxification, Disruption of hydrogen peroxide catabolic process
A0A182DWH4	1.8E−02	0.56	HLA-DRB3	HLA class II histocompatibility antigen, DR beta 3 chain OS = Homo sapiens OX = 9606 GN = HLA-DRB3 PE = 1 SV = 1	Impairment of antigen delivery to T cells and their activation, inability of T cells to proliferate and secrete cytokines which are normally involved in the immune response. Disruption of other parts of the immune response cascade, including B cells
P16455	2.6E−06	0.66	MGMT	Methylated-DNA–protein-cysteine methyltransferase OS = Homo sapiens OX = 9606 GN = MGMT PE = 1 SV = 1	Inability to replicate DNA
O95864	4.6E−02	0.67	FADS2	Fatty acid desaturase 2 OS = Homo sapiens OX = 9606 GN = FADS2 PE = 1 SV = 1	Dysfunction of the skin barrier and changes in the structure of the fat structure in the stratum corneum
P69905	2.5E−02	0.68	HBA1	Hemoglobin subunit alpha OS = Homo sapiens OX = 9606 GN = HBA1 PE = 1 SV = 2	Disruption of the catabolic processes of hydrogen peroxide, oxidation–reduction, oxygen delivery to various tissues, disruption of the cell death, and small molecule metabolic process
P68871	4.3E−02	0.70	HBB	Hemoglobin subunit beta OS = Homo sapiens OX = 9606 GN = HBB PE = 1 SV = 2	Disruption of the catabolic processes of hydrogen peroxide, oxidation–reduction, oxygen delivery to various tissues, disruption of the cell death, Disorder of small molecular metabolic process small molecule metabolic process, Dysregulation of nitric oxide biosynthetic process
Q9ULE0	8.0E−06	0.72	WWC3	Protein WWC3 OS = Homo sapiens OX = 9606 GN = WWC3 PE = 1 SV = 3	Increased cell proliferation and metastasis
O95969	3.2E−02	0.72	SCGB1D2	Secretoglobin family 1D member 2 OS = Homo sapiens OX = 9606 GN = SCGB1D2 PE = 2 SV = 1	Impaired inflammation modulation, tissue repair disorders and tumorigenesis, Disorder of cellular physiological process
O14556	1.6E−02	0.73	GAPDHS	Glyceraldehyde-3-phosphate dehydrogenase, testis-specific OS = Homo sapiens OX = 9606 GN = GAPDHS PE = 1 SV = 2	Increased glycolytic metabolism, tumor progression and metastatic proliferation of tumor cells, Disruption of the various membrane, cytoplasmic and nuclear transmissions

**Table 3:** Top 10 skin proteins with significant up-regulation in chemical warfare victims

Accession ID	*P*-value	Fold change	Gene name	Protein name	Pathogenesis pathway
P82932	1.8E−04	1.79	MRPS6	28S ribosomal protein S6, mitochondrial OS = Homo sapiens OX = 9606 GN = MRPS6 PE = 1 SV = 3	Disorder of oxidative phosphorylation process and disruption of mitochondrial protein synthesis, Decreased cell survival, impaired glycolic metabolism, and many biological activities
P05161	1.0E−02	1.62	ISG15	Ubiquitin-like protein ISG15 OS = Homo sapiens OX = 9606 GN = ISG15 PE = 1 SV = 5	Dysfunction of intracellular proteins and immune responses
Q9UDW1	1.9E−06	1.60	UQCR10	Cytochrome b-c1 complex subunit 9 OS = Homo sapiens OX = 9606 GN = UQCR10 PE = 1 SV = 3	Cell death and apoptosis, Production of reactive oxygen species (ROS)
Q969E8	2.5E−02	1.58	TSR2	Pre-rRNA-processing protein TSR2 homolog OS = Homo sapiens OX = 9606 GN = TSR2 PE = 1 SV = 1	Inhibition of NF-κB transcriptional activity and disruption of the apoptotic process
K7ELC2	7.4E−03	1.54	RPS15	40S ribosomal protein S15 OS = Homo sapiens OX = 9606 GN = RPS15 PE = 1 SV = 1	Loss of cell nucleus integrity and irregularities in cell growth and proliferation
P09669	1.8E−02	1.50	COX6C	Cytochrome c oxidase subunit 6C OS = Homo sapiens OX = 9606 GN = COX6C PE = 1 SV = 2	Irregularities in the process of apoptosis and oxidative phosphorylation of tissue cells
O95715	4.3E−03	1.49	CXCL14	C-X-C motif chemokine 14 OS = Homo sapiens OX = 9606 GN = CXCL14 PE = 1 SV = 2	Disruption of cell migration, impaired regulation of immune activity, especially anti-bacterial immunity, impaired use of CD14 + DC precursors and inflammation
P16403	3.5E−02	1.47	H1-2	Histone H1.2 OS = Homo sapiens OX = 9606 GN = HIST1H1C PE = 1 SV = 2	Disorders of cell cycle regulation, induction of apoptosis, the release of cytochrome C from mitochondria, and caspase-dependent cell death
Q99572	3.6E−02	1.46	P2RX7	P2X purinoceptor 7 OS = Homo sapiens OX = 9606 GN = P2RX7 PE = 1 SV = 4	Degradation of differentiated keratinocytes, release of pro-inflammatory mediators, proliferation of tumor cells, formation of reactive oxygen and nitrogen species
O14908	2.8E−03	1.45	GIPC1	PDZ domain-containing protein GIPC1 OS = Homo sapiens OX = 9606 GN = GIPC1 PE = 1 SV = 2	Impaired proliferation, cell surface polarity, cytokinesis and cell migration

Among the 129 proteins in this study that were associated with changes in statistically significant expression, their role and importance in the occurrence of some skin problems such as pruritus, inflammation, eczema, and melanoma observed in chemical warfare exposed to sulfur SM are different.

### GO and pathway enrichment analyses

**Fig. 2:**
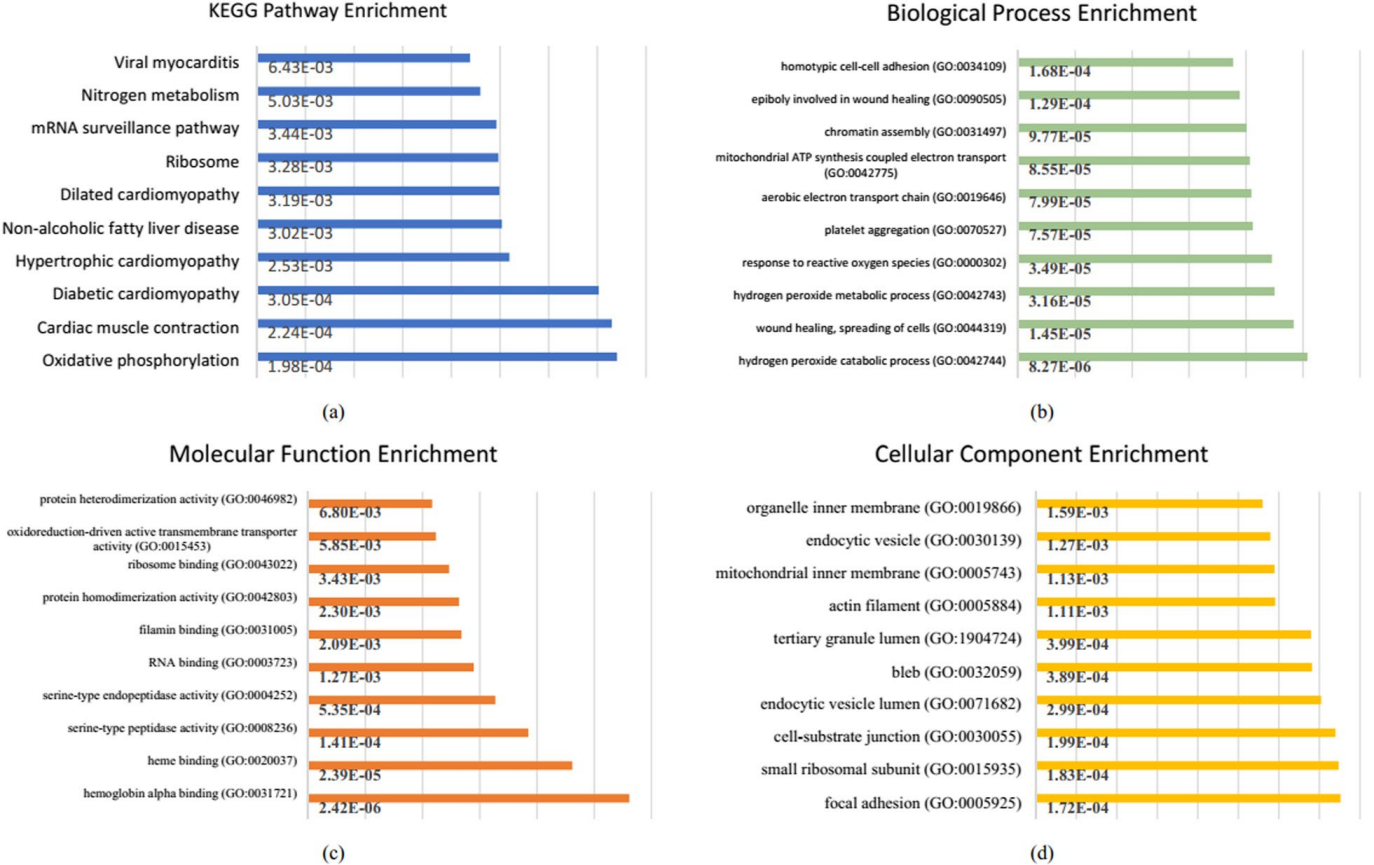
Top 10 GO and KEGG pathway enrichment analysis of the significant abundance protein’s skin

The list of GO analysis and the KEGG pathway are shown in Additional file 2. Figure 2 shows the top 10 enrichment analysis for all significant abundance proteins. The top 10 KEGG pathway of significant abundance proteins are found in Fig. 2a. In terms of BP, the top 10 significant abundance proteins are found in Fig. 2b, As far as MF is found in Fig. 2c. CC is found in Fig. 2d.

### PPI network and identification of hub genes

**Fig. 3:**
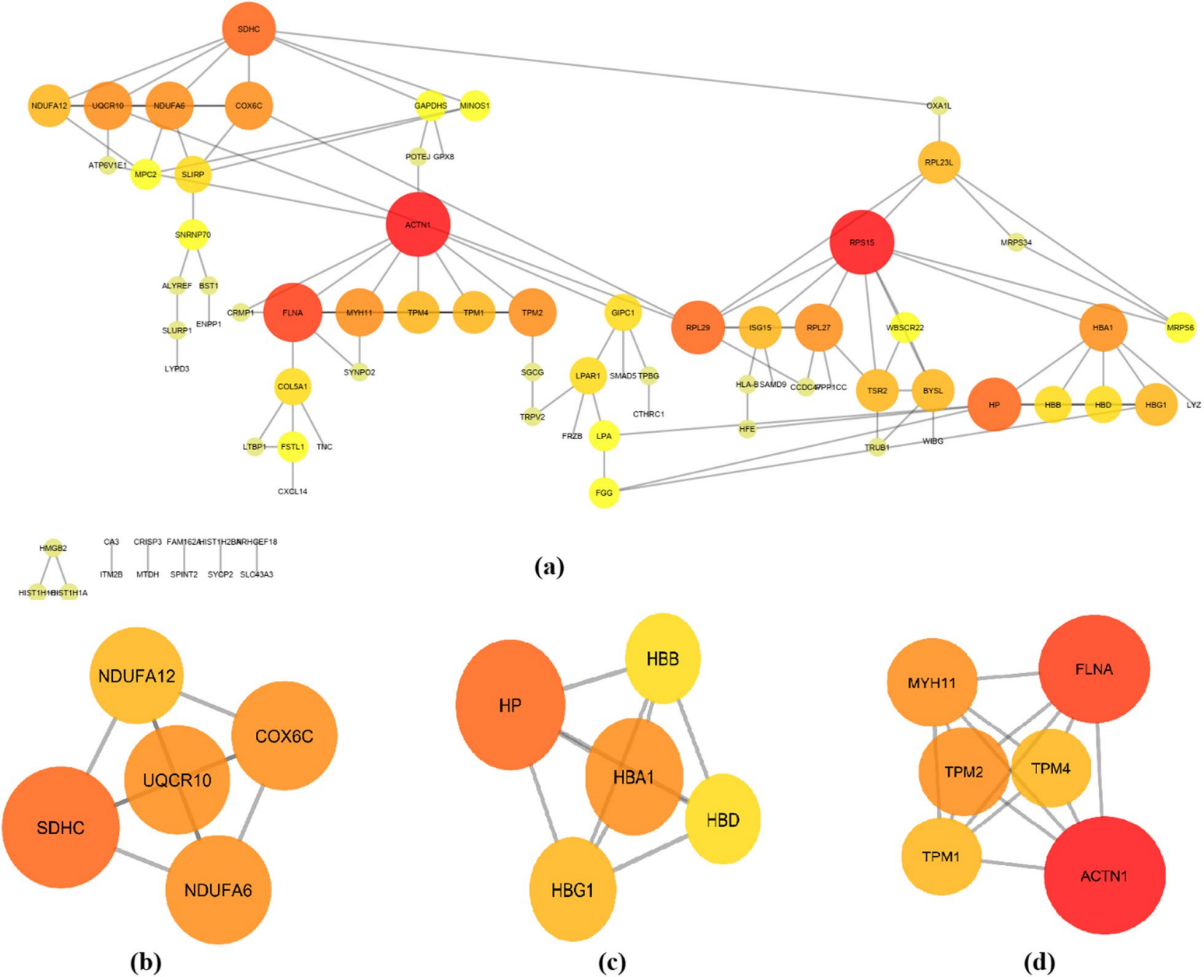
PPI networks of proteins and module analysis. (**a**); three parts of PPI network encompassed by t three modules filtered by app MCODE plugin in Cytoscape tool which were extracted from the PPI network (**b**, **c**, **d**)

A PPI network of the proteins was constructed using the STRING and Cytoscape tool. The PPI network contained 129 nodes, including 94 upregulated genes and 35 downregulated genes, and 121 edges; (Fig. 3a). The term “degree” in the PPI network means a number of interactions between two genes (nodes). The hub genes of the PPI network were filtered with a cut-off value of degree > 6. As a result, six hub genes were identified. The hub genes included *RPS15*, *ACTN1*, *FLNA*, *HP*, *SDHC*, *and RPL29*. Three modules were extracted from the PPI network, including module 3 with five nodes (i.e., NDUFA6, SDHC, DUFA12, COX6C, and UQCR10) (Fig. 3b), module 2 with five nodes included HBG1, HBB, HBD, HBA1, and HP (Fig. 3c), and module 1 with six nodes of TPM4, MYH11, ACTN1, TPM2, FLNA, and TPM1 (Fig. 3d).

## Discussion

Proteomic analysis of skin biopsy samples could aid in the discovery of potential disease biomarkers, as well as provide a better knowledge of disease pathophysiology. In the current study, TMT-based proteomics technologies on the skin of SM-exposed patients were used to show a different pattern compared to healthy controls. This is the first study to evaluate the skin's global proteome profiles in these patients in comparison with the control group.

Our findings showed six hub proteins (RPS15, ACTN1, FLNA, HP, SDHC, and RPL29) that three modules were extracted from the PPI network. Module 1 with five nodes includes NDUFA6, SDHC, DUFA12, COX6C, and UQCR10 (KEGG Pathway Enrichment: Oxidative phosphorylation, *p*-value: 9.3E-09 and FDR: 1.12E-07), module 2 with five nodes includes HBG1, HBB, HBD, HBA1, and HP, and module 3 with six nodes includes TPM4, MYH11, ACTN1, TPM2, FLNA, and TPM1 (KEGG Pathway Enrichment: Regulation of actin cytoskeleton, *p*-value: 0.001 and FDR: 0.004) were recovered from the PPI network.

Protein nodes in the first module are associated with mitochondrial electron transport chain (ETC) that includes 4 mitochondrial complexes. Significant alterations in the protein expression of mitochondrial respiratory chain complexes I, II, III, and IV in the SM-exposed patients compared to healthy controls are in line with previous studies [23–26]. There have been reports of mitochondrial dysfunction as a result of SM exposure [25, 26]. For example, Sourdeval et al. found that SM causes mitochondrial malfunction, which is followed by an increase in reactive oxygen species (ROS) formation and cellular OS [25]. Similar results were reported by Gould et al. for 2-chloroethyl ethylsulfide (CEES), and half mustard [26]. Another study found that mustards increase ROS production by changing cytochrome P450 activity by uncoupling microsomal electron transport at the flavoenzyme reductase [27]. Another mustard derivative, 11β: aniline mustard, alters the ETC at complex I, increasing ROS and OS generation while inhibiting oxygen consumption [28]. Another study reported chemical casualties had a higher pro-oxidant–antioxidant balance (PAB) score in comparison with the healthy control, indicating that they were exposed to OS [29].

ROS, particularly mitochondrial-derived ROS, play a role in a variety of signaling pathways, including eliciting antioxidative signaling, initiating DNA damage responses, affecting iron homeostasis, stimulating apoptosis, and signaling for cell survival and proliferation [30–32]. Thus, mitochondria may act as reservoirs of therapeutic targets in SM-exposed patients.

Altered expression and activity of mitochondrial complexes and increased OS in skin diseases similar to those exposed to mustard, including atopic dermatitis and atopic eczema, have also been reported. Previous research has found that nonlesional atopic dermatitis (ADNL) skin had higher expression of genes associated to OS [33]. Leman et al. discovered that increased complexes I and II activity in ADNL keratinocytes (KCs). Enhanced tricarboxylic acid cycle (TCA) turnover was discovered through metabolomics analysis. Mitochondria are important regulators of reactive oxygen species (ROS) production. In ADNL KCs, increased aerobic metabolism resulted in OS. ADNL human epidermal equivalents had a higher mitochondrial function and an increased OS response when compared with the controls. In addition, they discovered that glycolysis in ADNL KCs complements but does not replace mitochondrial metabolism. As a result, aerobic metabolism dominates in ADNL, causing OS [34].

Of note, patients participating in this study, in addition to skin complications caused by SM, also have respiratory problems and hypoxia. Hypoxia affects the mitochondrial shape, such as cristae structure, as well as protein expression, such as HIF proteins [35, 36]. HIF proteins regulate intermediate metabolism by inducing many target genes, such as raising the expression of glycolysis-related proteins and lowering oxygen-dependent pathways via modifying the ETC complex expression and activity [35] that is consistent with enhanced glycolysis and decreased tricarboxylic acid cycle (TCA) turnover was discovered through metabolomics analysis in SM-exposed patients [37, 38]. Subsequently, HIF-1 cooperates with STAT3 on the Haptoglobin (Hp) promoter to increase Hp gene expression (node in the second module). Hp is a significant acute phase protein that binds to extravascular hemoglobin (Hb) and eliminates the harmful Hb quickly via macrophages' CD163 scavenger receptor [39]. As a result, Hp maintain tissues from oxidative damage caused by Hb [40, 41]. Increased level of Hp was shown in plasma SM-exposed patients in comparison with the control group [42]. Patients with skin illnesses, such as psoriasis, have considerably higher Hp mRNA expression in epidermal keratinocytes than controls [43]. Kkeratinocyte-derived Hp may be involved in the skin's downregulation of inflammatory reactions.

In addition to ROS production, mitochondria also are involved within the function of melanocytes and pigments [44]. Hair disorders and pimples are a part of the wide selection of symptoms of mitochondrial disease. Skin problems including scaling, itching and erythema are reported in some mitochondrial diseases like mitochondrial encephalomyopathy and lactic acidosis [45].

Nodes (Tropomyosin 1, Tropomyosin 2, Tropomyosin 4, Myosin Heavy Chain 11, Filamin A and Actinin Alpha 1) in module 3 are important in the protection of the cellular cytoskeleton that all of them increased in SM-exposed patients compared with the control group. Studies showed that in the short term (several hours after SM exposure), changes in these proteins could cause blisters [46, 47], while increased level of these proteins several years after SM exposure is unclear. It can be due to ROS-induced apoptosis or cell proliferation [35].

In addition to the proteins of three modules mentioned above, some of the top 10 proteins were described in the following part. ISG15 proteins are induced by interferon type I and have many functions. It also acts as an extracellular cytokine and modifier of intracellular proteins [48]. Non-conjugated extracellular ISG15 acts as a cytokine to modulate immune responses [49, 50]. Inflammation-induced interferon type 1 can even appear as ulcerative skin lesions within the armpits, groin, and neck [51]. ISG15 appears to act as a carcinogenic protein as well as a tumor suppressor protein [52]. The presence of dermatitis and melanoma of the skin may not be unrelated to elevated levels of this protein. Previous studies have reported that TSR2 overexpression can suppress NF-қB transcription activity in Hep-2 cells [53]. Suppression of NF-κB induces apoptosis [54, 55]. In our study, a rise within the level of Pre-rRNA-processing protein TSR2 homolog was observed within the skin of chemical operations victims. Given the role that these proteins play in carcinogenesis and apoptosis, it will be expected that these proteins also are involved within the development of melanoma. RPS15 proteins, independent of their major role in ribosomal assembly and protein translation, also accomplish several extra-ribosomal functions, including regulating apoptosis, cell cycle arrest, cell proliferation and migration, invasion, and repair of DNA [56]. RPS15 may be associated with expression disorders in some sorts of cancer, including esophageal, lung and skin cancers [57–59]. In an exceedingly, Wang et al. indicated that RPS15 is over-expressed in gastric cancer tissues [60]. In our study, a rise in PRS15 ribosomal proteins was observed in chemical warfare victims exposed to SM. COX6C is found within the inner membrane of the mitochondria and is vital within the process of apoptosis [61]. Previous studies have shown that COX6C can cause chronic kidney disease (CKD), diabetes, carcinoma, melanoma and other diseases [62, 63]. Increased expression of COX6C in various invasive organs has been reported to always be observed in damaged tissue cells [64]. In our study, the level of COX6C proteins was significantly increased. Activation of P2RX7 stimulates several pathways, including the discharge of pro-inflammatory cytokines like IL-1 and IL-6, modulation of cell surface receptors, ROS and RNS formation and necrobiosis [65]. Immuno-histochemical studies indicate that P2RX7 within the upper layer of human skin is involved in the death of differentiated keratinocytes [66]. P2RX7 in Allergic dermatitis in humans is related to up-regulation within the basal epidermal layer of inflamed skin of patients with atopic eczema [67]. Increased P2RX7 levels are reported in human melanoma and in several melanoma cell lines [68]. In our study, we also observed a rise in the extent of P2RX7 protein within the skin of chemical operations victims. GIPC1 is involved in the transport of assorted membrane proteins and regulates various cellular processes including cell proliferation, cell surface polarity, cytokines and migration [69].

WWC3 proteins are involved in the regulation of cell proliferation and migration [70]. WWC3 proteins preventing the proliferation of malignant cells are capable of inducing the expression of apoptotic molecules. Within the presence of WWC3, the activity of caspase-3 and caspase-7 increases and WWC3 proteins act as an autophagy regulator in Non-Small Cell carcinoma. [71]. Down-regulation of WWC3, which is related to increased cell proliferation and high metastasis [72]. In our study, WWC3 proteins within the skin of chemical operations victims were associated with a significant down-regulation. MGMT is expressed as a repairing enzyme in any kind of human tissue, the quantity of which is expressed in several tissues. For instance, It has the highest expression within the liver; Excessive expression in epidermis, fibroblasts and melanocytes; It has relatively high within the lungs, kidneys and colon, and lowest within the pancreas, hematopoietic cells, lymph tissues, and brain [73, 74]. In animal models, increasing MGMT levels is to guard against the event of tumors including carcinoma [75, 76]. Therefore, a significant down-regulating MGMT protein in chemical operations victims is expected to be involved in the development of carcinoma. Delta-5-desachurase (FADS1) and delta-6-desachurase (FADS2) are two enzymes, which accountable for the synthesis of highly unsaturated fatty acids Linolic acid (LA) and Omega-6 fatty acid. Among the two essential fatty acids present within the skin, LA plays a crucial role in regulating the function of the skin barrier [77]. Atopic eczema is related to abnormal fatty acid metabolism, Because linolic acid (LA) deficiency in humans and rodents ends up in certain abnormalities within the skin of patients with atopic eczema [78]. In patients with atopic dermatitis, changes within the regulations of some transcription factors like SREBP-1c and PPAR-a have also been observed [79]. These transcription factors play a serious role in the regulation of FADS1 and FADS2 expressions [78]. In our study, FADS2 was related to a significant down-regulation within the skin of chemical operations victims.

## Conclusion

According to what has been observed from skin protein changes in chemical veterans exposed to SM, it can be concluded that the increase and decrease in expression of some proteins are significant with skin problems such as itching, scaling, eczema, and atopic dermatitis. Distinct proteins are associated with OS, inflammatory pathways, apoptosis, and cell proliferation.

## Supplementary Information

**Additional file 1**. The list of diffrentially abundant proteins.

**Additional file 2**. The list of GO analysis and the KEGG pathway.

## Data Availability

The datasets generated during and/or analysed during the current study are not publicly available due to confidentiality agreements, supporting data can only be made available to bona fide researchers subject to a non-disclosure agreement but are available from the corresponding author on reasonable request.

## References

[1] Evison D, Hinsley D, Rice P (2002). Chemical weapons. BMJ.

[2] Smith KJ (1995). Sulfur mustard: its continuing threat as a chemical warfare agent, the cutaneous lesions induced, progress in understanding its mechanism of action, its long-term health effects, and new developments for protection and therapy. J Am Acad Dermatol.

[3] Pechura CM, Rall DP. Dermatological effects of mustard agents and lewisite, in veterans at risk: the health effects of mustard gas and lewisite. National Academies Press (US); 1993. 25144024

[4] Balali-Mood M, Hefazi M (2005). The pharmacology, toxicology, and medical treatment of sulphur mustard poisoning. Fundam Clin Pharmacol.

[5] Momeni A-Z (1992). Skin manifestations of mustard gas: a clinical study of 535 patients exposed to mustard gas. Arch Dermatol.

[6] Smith W, Dunn M. Medical defense against blistering chemical warfare agents (Reannouncement with new availability information). Army Medical Research Inst. of Chemical Defense, Aberdeen Proving Ground; 1991.

[7] Pirie A (1947). The action of mustard gas on ox cornea collagen. Biochem J.

[8] Balali-Mood M, Hefazi M (2006). Comparison of early and late toxic effects of sulfur mustard in Iranian veterans. Basic Clin Pharmacol Toxicol.

[9] Emadi SN (2009). External urethral stenosis: a latent effect of sulfur mustard two decades post-exposure.

[10] Smith HW, Clowes GH, Marshall E (1919). On dichloroethyl sulfide (mustard gas). IV. The mechanism of absorption by the skin. J Pharmacol Exp Ther.

[11] Sabourin CL, Petrali JP, Casillas RP (2000). Alterations in inflammatory cytokine gene expression in sulfur mustard-exposed mouse skin. J Biochem Mol Toxicol.

[12] Rebholz B (2008). Role of NF-κB/RelA and MAPK pathways in keratinocytes in response to sulfur mustard. J Investig Dermatol.

[13] Kumar O, Sugendran K, Vijayaraghavan R (2001). Protective effect of various antioxidants on the toxicity of sulphur mustard administered to mice by inhalation or percutaneous routes. Chem Biol Interact.

[14] Black R, Read RW (1995). Biological fate of sulphur mustard, 1,1′-thiobis (2-chloroethane): identification of β-lyase metabolites and hydrolysis products in human urine. Xenobiotica.

[15] Feister AJ. Medical defense against mustard gas: toxic mechanisms and pharmacological implications. 1991.

[16] Kehe K (2009). Molecular toxicology of sulfur mustard-induced cutaneous inflammation and blistering. Toxicology.

[17] van Ooijen MP (2018). Identification of differentially expressed peptides in high-throughput proteomics data. Brief Bioinform.

[18] Xie Z (2021). Gene set knowledge discovery with enrichr. Curr Protoc.

[19] Kanehisa M (2021). KEGG: integrating viruses and cellular organisms. Nucleic Acids Res.

[20] Szklarczyk D (2021). The STRING database in 2021: customizable protein–protein networks, and functional characterization of user-uploaded gene/measurement sets. Nucleic Acids Res.

[21] Shannon P (2003). Cytoscape: a software environment for integrated models of biomolecular interaction networks. Genome Res.

[22] Bader GD, Hogue CWV (2003). An automated method for finding molecular complexes in large protein interaction networks. BMC Bioinform.

[23] Laskin JD (2010). Oxidants and antioxidants in sulfur mustard–induced injury. Ann N Y Acad Sci.

[24] Sabnam S (2020). CEES-induced ROS accumulation regulates mitochondrial complications and inflammatory response in keratinocytes. Chem Biol Interact.

[25] Sourdeval M (2006). Inhibition of caspase-dependent mitochondrial permeability transition protects airway epithelial cells against mustard-induced apoptosis. Apoptosis.

[26] Gould NS, White CW, Day BJ (2009). A role for mitochondrial oxidative stress in sulfur mustard analog 2-chloroethyl ethyl sulfide-induced lung cell injury and antioxidant protection. J Pharmacol Exp Therap.

[27] Brimfield A (2009). Free radical production from the interaction of 2-chloroethyl vesicants (mustard gas) with pyridine nucleotide-driven flavoprotein electron transport systems. Toxicol Appl Pharmacol.

[28] Fedeles BI (2011). Chemical genetics analysis of an aniline mustard anticancer agent reveals complex I of the electron transport chain as a target. J Biol Chem.

[29] Nobakht M. Gh BF (2016). Pro-oxidant–antioxidant balance in Iranian veterans with sulfur mustard toxicity and different levels of pulmonary disorders. Drug Chem Toxicol.

[30] Li Z (2017). Roles of reactive oxygen species in cell signaling pathways and immune responses to viral infections. Adv Virol.

[31] Redza-Dutordoir M, Averill-Bates DA (2016). Activation of apoptosis signalling pathways by reactive oxygen species. Biochimica et Biophysica Acta -Molecular Cell Research.

[32] Hanson ES, Leibold EA (1999). Regulation of the iron regulatory proteins by reactive nitrogen and oxygen species. Gene Expres J Liver Res.

[33] Blunder S (2018). Enhanced expression of genes related to xenobiotic metabolism in the skin of patients with atopic dermatitis but not with ichthyosis vulgaris. J Investig Dermatol.

[34] Leman G, et al. Mitochondrial activity is upregulated in nonlesional atopic dermatitis and amenable to therapeutic intervention. J Investig Dermatol. 2022. 10.1016/j.jid.2022.01.035 35341734

[35] Fuhrmann DC, Brüne B (2017). Mitochondrial composition and function under the control of hypoxia. Redox Biol.

[36] Plecitá-Hlavatá L, Ježek P (2016). Integration of superoxide formation and cristae morphology for mitochondrial redox signaling. Int J Biochem Cell Biol.

[37] Nobakht BF (2017). NMR spectroscopy-based metabolomic study of serum in sulfur mustard exposed patients with lung disease. Biomarkers.

[38] Ghoochani BFNM (2018). Metabolomics diagnostic approach to mustard airway diseases: a preliminary study. Iran J Basic Med Sci.

[39] Nielsen MJ, Moestrup SK (2009). Receptor targeting of hemoglobin mediated by the haptoglobins: roles beyond heme scavenging. Blood J Am Soc Hematol.

[40] Lim YK (2000). Haptoglobin reduces renal oxidative DNA and tissue damage during phenylhydrazine-induced hemolysis. Kidney Int.

[41] Lim S-K (1998). Increased susceptibility in Hp knockout mice during acute hemolysis. Blood.

[42] Mehrani H (2010). Plasma proteomic profile of sulfur mustard exposed lung diseases patients using 2-dimensional gel electrophoresis. Clin Proteom.

[43] Li P (2005). Localization of haptoglobin in normal human skin and some skin diseases. Int J Dermatol.

[44] Ni-Komatsu L, Orlow SJ (2007). Identification of novel pigmentation modulators by chemical genetic screening. J Investig Dermatol.

[45] Bodemer C (1999). Hair and skin disorders as signs of mitochondrial disease. Pediatrics.

[46] Mol MA, van den Berg RM, Benschop HP (2008). Proteomic assessment of sulfur mustard-induced protein adducts and other protein modifications in human epidermal keratinocytes. Toxicol Appl Pharmacol.

[47] Mol M, et al. Proteomics as a strategy to study the mechanistic toxicology of sulfur mustard. In: Proceedings of the 2003 Meeting of NA TO TGO04, Medicin Hat, Canada. 2003.

[48] Blomstrom DC (1986). Molecular characterization of the interferon-induced 15-kDa protein. Molecular cloning and nucleotide and amino acid sequence. J Biol Chem.

[49] Knight E, Cordova B (1991). IFN-induced 15-kDa protein is released from human lymphocytes and monocytes. J Immunol.

[50] Guilbaud G (2011). Evidence for sequential and increasing activation of replication origins along replication timing gradients in the human genome. PLoS Comput Biol.

[51] Martin-Fernandez M (2020). Systemic type I IFN inflammation in human ISG15 deficiency leads to necrotizing skin lesions. Cell Rep.

[52] Andersen J, Hassel B (2006). The interferon regulated ubiquitin-like protein, ISG15, in tumorigenesis: friend or foe?. Cytokine Growth Factor Rev.

[53] Pasparakis M (2009). Regulation of tissue homeostasis by NF-κB signalling: implications for inflammatory diseases. Nat Rev Immunol.

[54] Morais C (2006). Pyrrolidine dithiocarbamate exerts anti-proliferative and pro-apoptotic effects in renal cell carcinoma cell lines. Nephrol Dial Transplant.

[55] Morais C (2010). Inhibition of nuclear factor kappa B attenuates tumour progression in an animal model of renal cell carcinoma. Nephrol Dial Transplant.

[56] Anderson SJ (2007). Ablation of ribosomal protein L22 selectively impairs αβ T cell development by activation of a p53-dependent checkpoint. Immunity.

[57] Wang Q (2001). Cloning and characterization of full-length human ribosomal protein L15 cDNA which was overexpressed in esophageal cancer. Gene.

[58] Dang C (2006). Identification of dysregulated genes in cutaneous squamous cell carcinoma. Oncol Rep.

[59] Dmitriev AA (2012). Genetic and epigenetic analysis of non-small cell lung cancer with NotI-microarrays. Epigenetics.

[60] Wang H (2006). Overexpression of ribosomal protein L15 is associated with cell proliferation in gastric cancer. BMC Cancer.

[61] Belevich I, Verkhovsky MI (2008). Molecular mechanism of proton translocation by cytochrome c oxidase. Antioxid Redox Signal.

[62] Zaza G (2013). Downregulation of nuclear-encoded genes of oxidative metabolism in dialyzed chronic kidney disease patients. PLoS ONE.

[63] Masha RT, Houreld NN, Abrahamse H (2013). Low-intensity laser irradiation at 660 nm stimulates transcription of genes involved in the electron transport chain. Photomed Laser Surg.

[64] Tian B-X (2021). Differential expression and clinical significance of COX6C in human diseases. Am J Transl Res.

[65] Wiley J (2011). The human P2X7 receptor and its role in innate immunity. Tissue Antigens.

[66] Greig AV (2003). Purinergic receptors are part of a signaling system for keratinocyte proliferation, differentiation, and apoptosis in human fetal epidermis. J Investig Dermatol.

[67] Pastore S (2007). Stimulation of purinergic receptors modulates chemokine expression in human keratinocytes. J Investig Dermatol.

[68] White N, Butler PE, Burnstock G (2005). Human melanomas express functional P2X 7 receptors. Cell Tissue Res.

[69] De Vries L (1998). GIPC, a PDZ domain containing protein, interacts specifically with the C terminus of RGS-GAIP. Proc Natl Acad Sci.

[70] Wennmann DO (2014). Evolutionary and molecular facts link the WWC protein family to Hippo signaling. Mol Biol Evol.

[71] Han Q (2020). WW and C2 domain-containing protein-3 promoted EBSS-induced apoptosis through inhibiting autophagy in non-small cell lung cancer cells. J Thorac Dis.

[72] Hou J, Zhou J (2017). WWC3 downregulation correlates with poor prognosis and inhibition of Hippo signaling in human gastric cancer. Onco Targets Ther.

[73] Pieper RO (1997). Understanding and manipulating O6-methylguanine-DNA methyltransferase expression. Pharmacol Ther.

[74] Kalal BS, Upadhya D, Pai VR, Chemotherapy resistance mechanisms in advanced skin cancer. Oncol Rev. 2017;11(1). 10.4081/oncol.2017.326 PMC5379221 28382191

[75] Zaidi NH (1995). Transgenic expression of human MGMT protects against azoxymethane-induced aberrant crypt foci and G to A mutations in the K-ras oncogene of mouse colon. Carcinogenesis.

[76] Shiraishi A, Sakumi K, Sekiguchi M (2000). Increased susceptibility to chemotherapeutic alkylating agents of mice deficient in DNA repair methyltransferase. Carcinogenesis.

[77] Hartop P, Prottey C (1976). Changes in transepidermal water loss and the composition of epidermal lecithin after applications of pure fatty acid triglycerides to the skin of essential fatty acid-deficient rats. Br J Dermatol.

[78] Nakamura MT, Nara TY (2004). Structure, function, and dietary regulation of Δ6, Δ5, and Δ9 desaturases. Annu Rev Nutr.

[79] Schlotter YM (2009). Altered expression of fatty acid desaturases in the skin of dogs with atopic dermatitis. J Dermatol Sci.

